# Transcriptomic analysis of benznidazole-resistant and susceptible *Trypanosoma cruzi* populations

**DOI:** 10.1186/s13071-023-05775-4

**Published:** 2023-05-22

**Authors:** Davi Alvarenga Lima, Leilane Oliveira Gonçalves, João Luís Reis-Cunha, Paul Anderson Souza Guimarães, Jeronimo Conceição Ruiz, Daniel Barbosa Liarte, Silvane Maria Fonseca Murta

**Affiliations:** 1 Genômica Funcional de Parasitos, Instituto René Rachou (IRR/Fiocruz Minas), Av. Augusto de Lima 1715, Belo Horizonte, MG CEP 30190-002 Brazil; 2 Informática de Biossistemas, Genômica e Bioengenharia, Instituto René Rachou (IRR/Fiocruz Minas), Belo Horizonte, MG Brazil; 3 Department of Biology, University of York, York, UK; 4 Departamento de Biologia, Universidade Federal do Piauí, Teresina, PI Brazil

**Keywords:** RNAseq, Transcriptomics, Chagas disease, *Trypanosoma cruzi*, Resistance, Benznidazole

## Abstract

**Background:**

Chagas disease (CD), caused by the parasite *Trypanosoma cruzi*, is a serious public health concern in Latin America. Nifurtimox and benznidazole (BZ), the only two drugs currently approved for the treatment of CD, have very low efficacies in the chronic phase of the disease and several toxic side effects. *Trypanosoma cruzi* strains that are naturally resistant to both drugs have been reported. We performed a comparative transcriptomic analysis of wild-type and BZ-resistant *T. cruzi* populations using high-throughput RNA sequencing to elucidate the metabolic pathways related to clinical drug resistance and identify promising molecular targets for the development of new drugs for treating CD.

**Methods:**

All complementary DNA (cDNA) libraries were constructed from the epimastigote forms of each line, sequenced and analysed using the Prinseq and Trimmomatic tools for the quality analysis, STAR as the aligner for mapping the reads against the reference genome (*T. cruzi* Dm28c—2018), the Bioconductor package EdgeR for statistical analysis of differential expression and the Python-based library GOATools for the functional enrichment analysis.

**Results:**

The analytical pipeline with an adjusted *P*-value of < 0.05 and fold-change > 1.5 identified 1819 transcripts that were differentially expressed (DE) between wild-type and BZ-resistant *T. cruzi* populations. Of these, 1522 (83.7%) presented functional annotations and 297 (16.2%) were assigned as hypothetical proteins. In total, 1067 transcripts were upregulated and 752 were downregulated in the BZ-resistant *T. cruzi* population. Functional enrichment analysis of the DE transcripts identified 10 and 111 functional categories enriched for the up- and downregulated transcripts, respectively. Through functional analysis we identified several biological processes potentially associated with the BZ-resistant phenotype: cellular amino acid metabolic processes, translation, proteolysis, protein phosphorylation, RNA modification, DNA repair, generation of precursor metabolites and energy, oxidation–reduction processes, protein folding, purine nucleotide metabolic processes and lipid biosynthetic processes.

**Conclusions:**

The transcriptomic profile of *T. cruzi* revealed a robust set of genes from different metabolic pathways associated with the BZ-resistant phenotype, proving that *T. cruzi* resistance mechanisms are multifactorial and complex. Biological processes associated with parasite drug resistance include antioxidant defenses and RNA processing. The identified transcripts, such as ascorbate peroxidase (APX) and iron superoxide dismutase (Fe-SOD), provide important information on the resistant phenotype. These DE transcripts can be further evaluated as molecular targets for new drugs against CD.

**Graphical Abstract:**

**Figure:**
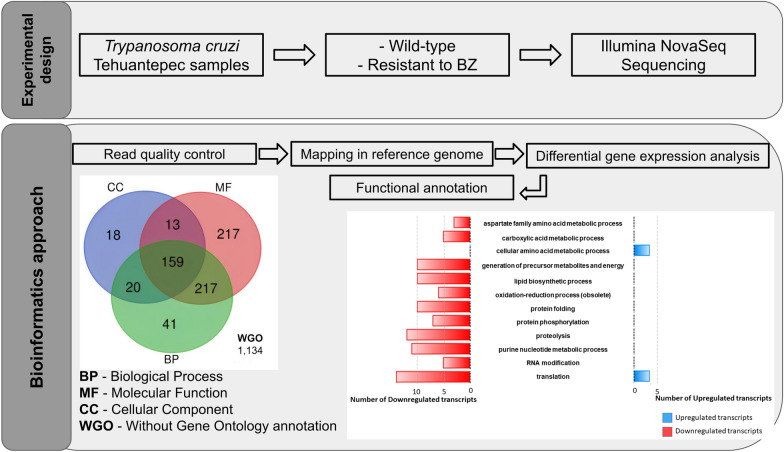

**Supplementary Information:**

The online version contains supplementary material available at 10.1186/s13071-023-05775-4.

## Background

Chagas disease (CD) is caused by the protozoan parasite *Trypanosoma cruzi*, with current estimates of more than 6 million infections worldwide [1]. Although endemic in Latin American countries, the increased movement of people between countries due to globalisation has led to CD also impacting European and North American countries [2]. The disease presents in two clinically distinct phases: acute and chronic. Only two drugs, benznidazole (BZ) and nifurtimox (NFX), are currently approved for clinical use, but both have low cure rates during the chronic stage of the disease and are associated with serious side effects that may result in treatment interruption [3]. Differences in the susceptibility of *T. cruzi* strains to BZ and NFX may explain, at least in part, the low cure rates observed in patients treated for CD [4–6]. Thus, the identification of genes that are differentially expressed (DE) in the BZ-resistant *T. cruzi* population would improve our knowledge of the molecular mechanism underlying the drug-resistant phenotype.

The nitroheterocyclic compounds BZ and NFX are pro-drugs that require activation by nitroreductases for inducing reactive oxygen species formation, which causes toxicity in parasites by binding to the DNA, RNA, lipids, proteins and low-molecular-weight thiols [7–9]. Functional analysis has revealed reduced levels of nitroreductase type I (NTR-1) in *T. cruzi* and *T. brucei* to be associated with resistance to nitroheterocyclic compounds, whereas overexpression of this enzyme results in hypersensitivity [9, 10]. In our previous studies, we showed that antioxidant defense enzymes, such as iron superoxide dismutase (Fe-SOD), tryparedoxin peroxidase and ascorbate peroxidase (APX) are expressed at higher levels in the *T. cruzi* population with in vitro-induced resistance to BZ [11–13].

The sequencing of trypanosomatid genomes using new-generation technologies has significantly improved our understanding of the underlying metabolic processes in these parasites**.** Several phenotypes have been investigated by applying this technique in both the host cell and parasite. RNA sequencing (RNAseq) studies have demonstrated remodelling of the transcript profile of *T. cruzi* and human fibroblasts during intracellular infection [14]. Parasite transcript modifications are crucial for the establishment of infection, interaction with host cells and modifications of the dynamic expression of immune response genes and cell cycle regulators. Comparative transcriptome analysis of fibroblasts infected with *T. cruzi* strains Sylvio and Y showed a strong relationship with host-parasite interactions [15]. In another study, slight temperature changes increased cell death in metacyclic trypomastigotes due to the deregulation of genes involved in essential processes of *T. cruzi* [16]. Comparative transcriptome analysis of virulent (CL Brener) and non-virulent (CL-14) *T. cruzi* strains revealed delayed expression of genes encoding surface proteins associated with the transition from amastigotes to trypomastigotes in the CL-14 strain [17]. Transcriptomic analysis of the three main life-cycle forms of *T. cruzi* revealed that protein surface remodelling and metabolic switches are the basis for parasite differentiation [18]. Comparative transcriptome analysis of susceptible and BZ-resistant *T. cruzi* clones using pyrosequencing methodology identified 133 DE transcripts in the BZ-resistant *T. cruzi* clone [19]. However, the coverage obtained was 5×, limiting statistical analysis for gene identification. Hence, the genomic-scale alteration in expression that results in BZ resistance remains unknown. In the present study, we used RNAseq methodology with an average coverage of 275× to compare the transcriptome of BZ-resistant (17LER) and wild-type (17WTS) *T. cruzi* lines derived from the Tehuantepec strain and identify DE transcripts and metabolic pathways potentially associated with the BZ-resistant phenotype.

## Methods

### *Trypanosoma cruzi* populations

The *T. cruzi* lines used in this study were the BZ-resistant *T. cruzi* population (17LER), derived from the Tehuantepec cl2 susceptible wild-type strain (Tc I), namely 17WTS [20], provided by Philippe Nirdé (Génétique Moléculaire des Parasites et des Vecteurs, Montpellier, France). The BZ-resistant *T. cruzi* population (17LER) was obtained in vitro by increasing BZ concentration in a stepwise manner. The final 17LER parasites obtained were resistant to a BZ dose of 220 µM and thereby were 20-fold less susceptible to BZ than their wild-type counterparts of the 17WTS population (11 µM) [20]. In addition, the resistance index was maintained even after differentiation into amastigotes, and the BZ-resistant phenotype was stable even after 6 months of culture without drug pressure [20].

Epimastigote forms from both populations were cultured as described by Nogueira et al. [11]. Three independent biological replicates of each population were cultured under drug pressure. At the logarithmic phase of growth, the parasites were washed with phosphate buffered saline and centrifuged, following which the sediment was extracted and frozen at − 70 °C for subsequent RNA extraction.

### RNAseq library preparation and sequencing

Total RNA from *T. cruzi* samples was extracted using TRIzol reagent according to the manufacturer's protocol (Invitrogen, Thermo Fisher Scientific, Waltham, MA, USA), then a new RNA was extracted and purified according to the manufacturer's instructions using an RNA extraction kit (RNeasy; Qiagen, Hilden, Germany) to increase the purity of the samples. The total RNA concentration was determined using a fluorescence assay in Qubit (Thermo Fisher Scientific). The quality and integrity of the total RNA extracted from the biological replicates of each *T. cruzi* population sample (17WTS and 17LER) were evaluated using the Bioanalyzer 2100 system (Agilent Technologies, Inc., Santa Clara, CA, USA) and subjected to complementary DNA (cDNA) synthesis.

Six libraries were constructed based on the poly A tail capture protocol using the NEBNext® UltraTM II RNA Library Prep Kit library construction kit (Illumina, Inc., San Diego, CA, USA) and 10 μg of total RNA for each library. The samples were sequenced by Novogene (Hong Kong, China) using NovaSeq technology® (Illumina Inc.) based on the sequencing of 150-bp paired-end fragments, by following the manufacturer’s standard protocols.

### Genome data

*Trypanosoma cruzi* Dm28c—2018 genome data were downloaded from the TritrypDB database version 46 [21]. This genome version refers to the sequencing of the Dm28c strain of *T. cruzi* that uses a combination of long-read (PacBio) and short-read (HiSeq) sequencing approaches. The total size of the assembly was 53.2 Mb, which is compatible with the haploid genome of the parasite. The functional annotation of this genome was carried out based on annotations from the TriTrypDB online resource and the Conserved Domain Database (CDD). Approximately 17,000 protein-coding genes were identified, in addition to retrotransposons and tandem repeats [22].

### Data quality control

The raw sequence reads in FASTQ format were evaluated in terms of read quality (per base sequence quality, per base G + C content, sequence length distribution and low complexity sequences) using the Prinseq program version 0.20.4 [23]. Trimmomatic version 0.22 [24] was used to filter and trim the sequences. The adapter sequences, reads shorter than 100 bp and low-quality reads based on the PHRED score (mean Q < 30) were identified. Only those reads with both pairs above the cut-offs were retained.

STAR software [25] version 2.7.7a was used to map the reads in the reference genome, and a curated general feature format (GFF) obtained from the same database was used to guide the alignment process, allowing up to three mismatches per read.

### Differential expression analysis

HTseq-count software version 0.5.4p3 [26] with the union model was used to count the total number of mapped reads for each annotated gene in the GFF file.

The counting matrix generated was used for differential expression analysis using the EdgeR package version 3.28.1 [27, 28] developed in the R programming language version 3.6.3 ® Foundation for Statistical Computing, Vienna, Austria). For the differential expression analysis, the transcripts with low counts (counts per million [CPM] < 1) in at least two biological replicates were removed to avoid analysing transcripts without statistical support. In addition, transcripts with an adjusted *P-*value of < 0.05 and fold-change (FC) ≥ 1.5 were set as thresholds to define DE genes.

### Functional analysis

After the differential gene expression analysis, a search for relevant domains and sites was performed using Interproscan to set a description of those identified transcripts described as hypothetical or unknown functions.

The GOATools package version 1.2.3, developed in Python 3, was used to calculate the functional enrichment analysis by overrepresentation using the upregulated and downregulated transcripts in comparison with the parasite reference transcriptome [29]. Functional analysis of the transcripts was based on Gene Ontology (GO) vocabulary. The category with the most informative GO considered for our analysis was "biological processes". An adjusted *p*-value < 0.05 generated using statistical analysis performed by GOATools was set as the threshold to define the functional enrichment significance. The GO data of the transcripts of *T. cruzi* used as a reference were the Gene Association File (GAF), available in TriTrypDB (https://tritrypdb.org/tritrypdb/app) version 46 [21]. Redundant functional categories were manually filtered, and the most specific category was retained for further analysis. Non-specific GO terms containing the same transcripts associated with another GO were removed. In addition, transcripts enriched only in non-specific categories were reallocated to other more informative categories based on the TriTrypDB data to favour the interpretation of the results.

## Results

### Overview of sample sequencing

We compared the transcriptomes of BZ-resistant (17LER) and wild-type (17WTS) *T. cruzi* populations. cDNA libraries were constructed, sequenced and analysed for identifying the DE transcripts associated with resistance to BZ.

The following parameters were evaluated using the read-quality analysis: (i) quantity of the sequenced reads; (ii) minimum and maximum sizes; and (iii) percentage of guanine–cytosine content in the samples. Approximately 82% of the reads were retained for the subsequent steps. The samples were mapped to the reference genome (Dm28c—2018 *T. cruzi* strain), resulting in an average coverage of 275× for the Tehuantepec *T. cruzi* strain. Six replicates generated 345,731,468 reads of approximately 150 bp each. Additional file 1: Table S1 summarises the characteristics of the reads that remained after the removal of low-quality data.

The data in Additional file 1: Table S1 show that each sample had approximately 22 million associated reads in both populations and that there were no major differences between the investigated groups. Tehuantepec reads had 130,247,626 (91.7%) reads mapped in the reference genome, with approximately 39 million reads (27.8%) mapped in multiple copy regions.

**Fig. 1:**
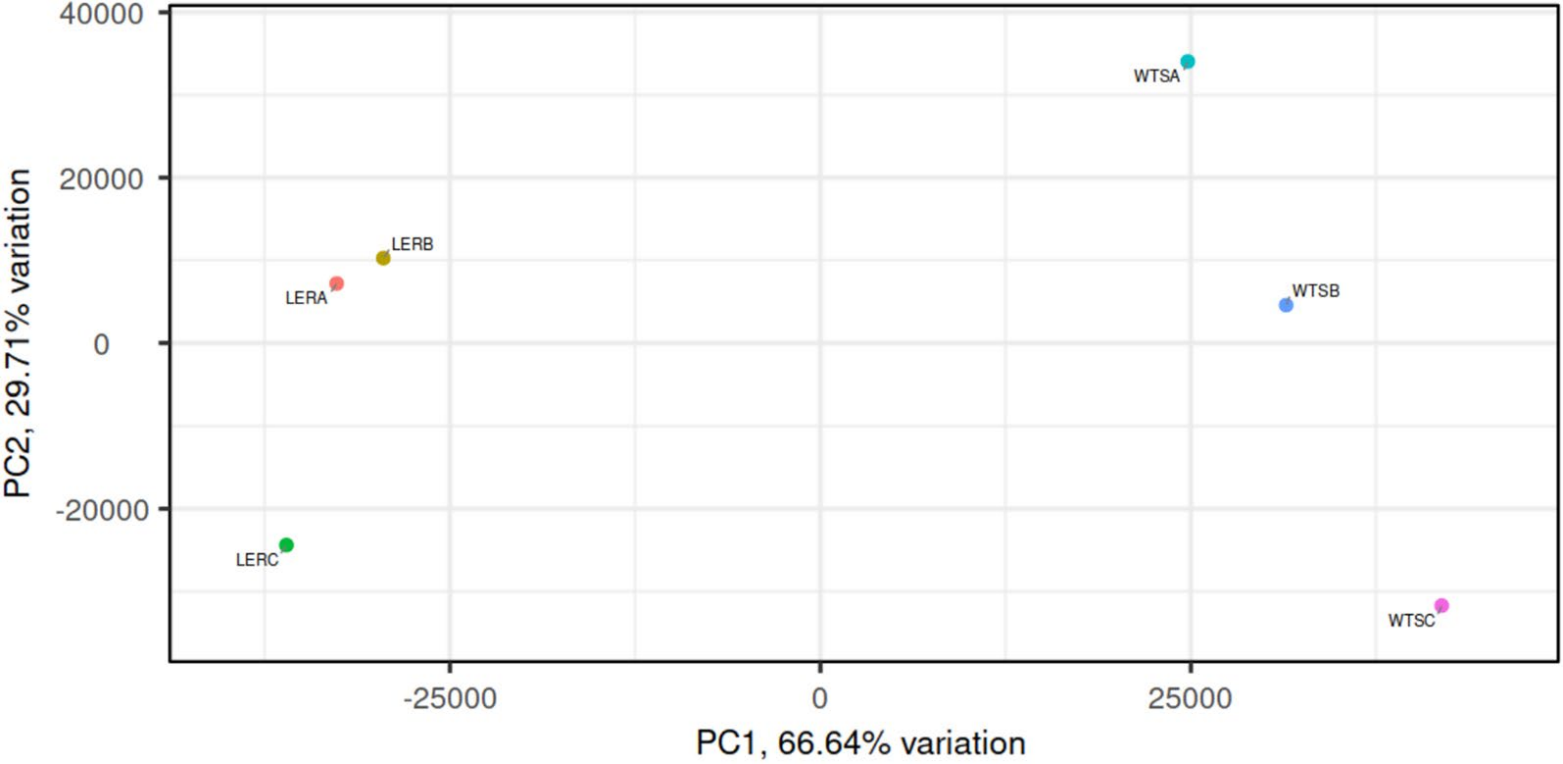
Principal component analysis from benznidazole (BZ)-resistant and wild-type *Trypanosoma cruzi* populations. The graph shows six replicates used in this study: wild-type sample A (WTSA) (cyan), wild-type sample B (WTSB) (blue) and wild-type sample C (WTSC) (pink) replicates represent wild-type samples; BZ-resistant sample A (LERA) (red), BZ-resistant sample B (LERB) (brown) and BZ-resistant sample C (LERC) (green) represent BZ-resistant samples. The distribution of samples represents the differences between the samples. Analysis of the graph reveals that the wild-type and BZ-resistant populations are clearly distinguished along the* X*-axis. PC, Principal component

Principal component analysis (PCA) was performed to assess the differences between samples based on the number of reads for each transcript. All transcripts with insufficient read counts in ≥ 2 replicates were excluded from the analysis. A PCA plot with the distribution of the samples around the principal component axes is shown in Fig. 1.

By generating a graph based on the PCA results, we were able to summarise approximately 95% of the data variation in the two principal components (Fig. 1). The* X*- and* Y*-axis components represent 66.6% and 29.7% of the data variation, respectively. The distribution on the* Y*-axis showed a variation between biological replicates expected based on the *T. cruzi* plasticity reported in the literature, whereas component 1 (*X*-axis) showed sufficient variation to distinguish samples from the susceptible and BZ-resistant *T. cruzi* populations.

### Differential expression analysis

**Fig. 2:**
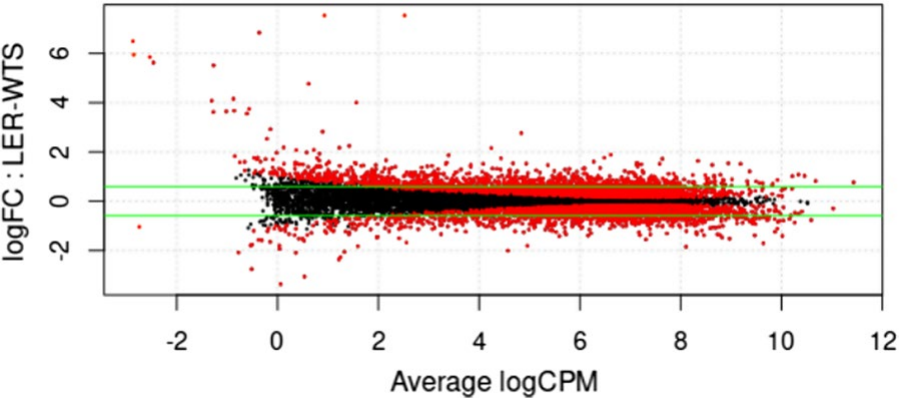
Expression plot comparing *T. cruzi* wild-type (17WTS) and benznidazole-resistant (17LER) RNA transcript levels. The CPM is represented on the *X*-axis, and the LogFC is represented on the *Y*-axis. Red and black dots represent transcripts with an adjusted *P*-value < 0.05 and > 0.05, respectively. The green lines represent the minimum FC value for the selected differentially expressed transcripts (Log_2_FC = 0.59). LogCPM, logarithm counts per million; LogFC, logarithm of the fold-change

After the samples were represented in a PCA, we performed a differential expression evaluation using a single comparison group, comparing the 17LER population with the 17WTS population. The expression plot shown in Fig. 2 reveals a close relationship between the average number of LogCPMs and the adjusted* P*-value for transcripts, suggesting that a higher number of counts implies a higher level of significance to the data. Transcripts with a lower number of associated read counts or multi-mapping regions may have high FC values owing to the low number of counts, as shown in Fig. 2 for transcripts with logCPM values < 3. The green horizontal lines in Fig. 2 mark the Log_2_FC = 0.59 (FC > than 1.5), which is considered to be the cut-off point to mark the transcripts used in the functional enrichment analysis.

Differential expression analysis identified 12,150 transcripts with an adjusted *P*-value ≤ 0.05. From the total number of identified transcripts, 1819 (15%) were considered to be DE with an adjusted *P*-value < 0.05 and FC > 1.5. Of these 1819 DE transcripts, 1067 (58.7%) were upregulated and 752 (41.3%) were downregulated in the BZ-resistant *T. cruzi* population. Regarding functional annotation, of the 1819 DE transcripts identified, 1522 (83.7%) presented functional annotations and 297 (16.3%) were assigned as hypothetical proteins without predicted function. In the functional enrichment analysis, a section was used with the transcripts whose expression (FC) was between 1.5 and 10; below this range, the difference was considered to indicate low expression, whereas above it may represent the sequencing of repetitive regions. Additional file 2: Figure S1 shows the sections into which the data were divided and the range in which functional enrichment was performed (represented in green).

### Functional analysis of DE transcripts

**Fig.3:**
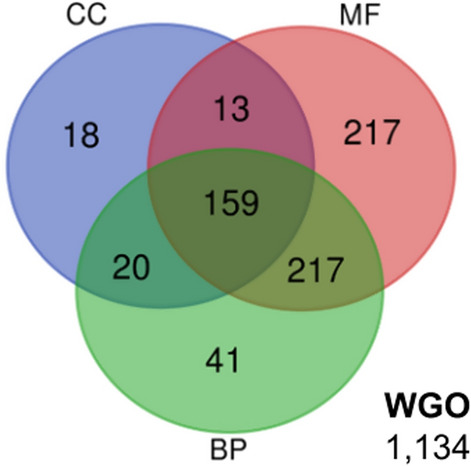
Venn diagram of shared and specific Gene Ontology (GO) terms for the differentially expressed (DE) transcripts. In total, 1819 DE transcripts (fold-change ≥ 1.5 and adjusted *P*-value < 0.05) of benznidazole-resistant *T. cruzi* were compared and grouped together using the GO categories biological process (BP), molecular function (MF) and cellular component (CC). WGO represents the GO category of transcripts without assigned GO group. Numbers refer to the total number of shared and specific sequences in each ontology group

The 1819 DE transcripts (1067 upregulated and 752 downregulated) were subjected to functional enrichment analysis and compared with the complete reference genome annotations. A group of 685 of these 1819 DE transcripts (37.7%) had associated GO terms. The distribution of functionally analysed transcripts according to the GO analysis present in the annotation file is shown in the Venn diagram (Fig. 3).

### Transcripts upregulated in the BZ-resistant *T. cruzi* line

Functional enrichment analysis revealed GO biological process categories that were significantly more or less frequent in a “test group” (DE transcripts) compared with a “reference group” (transcriptome) (*T. cruzi* Dm28c predicted on proteome) (Table 2). Functional enrichment analysis identified the upregulated transcripts as belonging to 34 functional enriched GO categories, with 14 upregulated transcripts being in the biological process (BP) GO category, 14 in the molecular function (MF) GO category and six in the cellular component (CC) GO category. The data obtained from GO terms MF and CC categories provide information on molecular interactions and cellular localisation, respectively. The BP category presents well-characterised mechanisms that help infer biological mechanisms important for *T. cruzi* resistance to BZ. Based on these results, we focused further analysis on the BP category.

Of the 1067 (58.7%) upregulated transcripts, including those with functional annotation and hypothetical proteins identified in the BZ-resistant *T. cruzi* line, 174 (15.25%) did not present any GO term associated with biological processes (15 enriched and 159 without enrichment), 118 (11.1%) transcripts did not present any GO terms associated with on biological processes and 775 (72.6%) transcripts with functional annotation and hypothetical proteins did not present any associated GO terms (Table 1).

**Table 1:** Transcripts differentially expressed between wild-type and benznidazole-resistant *Trypanosoma cruzi* populations and Gene Ontology functional enrichment analysis

DE transcripts	GO category			Without GO category (*n*)	Total (*n*)
	Biological process		Cellular component or molecular function (*n*)		
	Enriched (*n*)	Not enriched (*n*)			
*Functional annotation*^*a*^					
Upregulated	15	159	118	609	901
Downregulated	251	12	130	228	621
*Hypothetical protein*^*b*^					
Upregulated	0	0	0	166	166
Downregulated	0	0	0	131	131
*Total number of DE transcripts identified*	266	171	248	1134	1819

*DE* Differentially expressed,* GO* Gene Ontology

^a^Transcripts with functional annotation

^b^Transcripts with no assigned function

The most representative terms for the upregulated transcripts were cellular amino acid metabolic processes and translation (Table 2). These data are representative of the complete results (Additional file 3: Table S2) and suggest that categories marked as enriched among transcripts can contribute to the BZ-resistant phenotype.

**Table 2:** Upregulated enriched transcripts associated to the Gene Ontology biological process category

ID	Description	Fold-change	*P*-value
*Cellular amino acid metabolic process*			
C4B63_42g164	Tyrosine Aminotransferase^a^	3	8E-04
C4B63_24g246	Kynureninase^a^	1.5	7E-06
C4B63_462g12	Glutamamyl Carboxypeptidase^a^	2.7	2E-07
*Translation*			
C4B63_255g16	RNA Polymerase IIA Largest Subunit	1.7	5E-22
C4B63_46g417c	DNA-Directed RNA Polymerase I Subunit	1.5	4E-10
C4B63_52g36	50S Ribosomal Protein L16	1.9	4E-28

See Additional file 3: Table S2 for complete data

^a^Transcripts with > 1 copy in the corresponding Gene Ontology category

Table 2 shows the non-redundant and more specific categories to be highlighted in the analysis using the criterion of specificity and distance between the branches of the GO tree. Twelve transcripts upregulated in the BZ-resistant *T. cruzi* line were grouped in the cellular amino acid metabolic process category (GO: 0006519). This group included two kynureninases (1.5-fold upregulated), three tyrosine aminotransferases (1.7- to 3-fold upregulated) and seven glutamamyl carboxypeptidases (1.9- to 2.7-fold upregulated). In the translation (GO: 0006412) category, three transcripts were upregulated in the same line: RNA polymerase IIA (1.7-fold upregulated), DNA-directed RNA polymerase I subunit (1.5-fold upregulated) and 50S ribosomal protein L16 (1.9-fold upregulated). Transcripts encoding glutamamyl carboxypeptidase were enriched in a more specific category of amino acid metabolism: the arginine metabolic process. The two identified categories are related because amino acid metabolism generates several metabolites that are used in the translation process.

### Transcripts downregulated in the BZ-resistant *T. cruzi* line

Functional enrichment analysis was performed, and 144 functional categories of GO enriched for the downregulated transcripts were identified, of which 96 were for BP, 28 for MF and 20 for CC. The most representative GO terms were protein folding, oxidation–reduction, protein phosphorylation, lipid biosynthetic process and RNA modification and translation. The enrichment of the downregulated transcripts is presented in Table 3. Due to the large number of functional categories identified and their redundancy, only the most specific transcripts that were not repeated in other categories were retained for overview.

**Table 3:** Downregulated enriched transcripts associated to the Gene Ontology biological process category

ID	Description	Fold-change	*P*-value
*Protein folding*			
C4B63_2g690	Chaperonin Alpha Subunit^a^	2.1	1.2E−56
C4B63_81g100	Chaperonin Hsp60, Mitochondrial Precursor^a^	2.2	4.1E−06
C4B63_42g42	Co-Chaperone Grpe	2.1	9.1E−48
C4B63_18g293	Heat Shock 70 KDa Protein, Mitochondrial Precursor^a^	2.6	2.4E−82
C4B63_8g2652c	Heat Shock Protein 10 kDa^a^	1.5	6.4E−10
C4B63_2g430	Heat Shock Protein	1.8	1.4E−41
C4B63_175g9	Heat Shock Protein DNAJ^a^	2	2.0E−42
C4B63_17g90	T-Complex Protein 1, Beta/Delta Subunit^a^	1.6	8.9E−31
*Obsolete oxidation–reduction process*			
C4B63_34g109	Glutaredoxin	2.2	1.5E−19
C4B63_429g1	Glycerol-3-Phosphate Dehydrogenase, NAD-Dependent, N-Terminal	2.2	8.4E−04
C4B63_220g12	Peroxidoxin^a^	1.7	6.3E−18
C4B63_27g162	Peroxisomal Biogenesis Factor 11	2.1	7.5E−36
C4B63_18g258	Superoxide Dismutase	2	4.4E−05
C4B63_25g294	Thioredoxin-Like	2.5	9.9E−91
*Protein phosphorylation*			
C4B63_27g189	Cdc2-Related Kinase 12	1.7	2.2E−28
C4B63_23g248	Inorganic Pyrophosphatase	1.7	3.9E−19
C4B63_27g204	Kinetoplastid Kinetochore Protein 10	1.6	1.3E−16
C4B63_157g34	Protein Kinase^a^	4.6	3.1E−11
C4B63_60g133	Protein Tyrosine Phosphatase-Like Protein	1.8	1.2E−30
C4B63_163g32	Rac Serine-Threonine Kinase	1.5	7.5E−03
C4B63_208g28	Tousled-Like Kinase II	1.5	3.1E−05
*Proteolysis*			
C4B63_40g46	ATP-Dependent Protease Subunit HslV	1.8	6.4E−35
C4B63_157g33	ATP-Dependent Zinc Metallopeptidase^a^	3.7	1.7E−06
C4B63_27g181	Cysteine Peptidase, Clan CA, Family C19	1.5	8.1E−20
C4B63_16g99	Cytosolic Leucyl Aminopeptidase^a^	1.5	1.6E−15
C4B63_12g153	Metallo-Peptidase, Clan MF, Family M17	1.7	7.4E−36
C4B63_20g363	Mitochondrial ATP-Dependent Zinc Metallopeptidase	1.5	2.0E−16
C4B63_122g16	Oligopeptidase B	2	7.1E−62
C4B63_7g423	Proteasome Beta 7 Subunit	1.6	1.7E−20
C4B63_98g16	Surface Protease GP63^a^	1.6	1.4E−08
C4B63_37g366	Thimet Oligopeptidase^a^	2.3	4.1E−03
C4B63_235g5	Ubiquinone Biosynthesis Protein-Like Protein	1.7	1.3E−08
C4B63_252g9	Ubiquitin Carboxyl-Terminal Hydrolase	1.6	3.9E−03
*Generation of precursor metabolites and energy, glycolytic process*			
C4B63_54g173	Cytochrome B-C1 Complex Subunit 7	1.5	4.4E−23
C4B63_54g23	Enoyl-CoA Hydratase/Isomerase Family Protein	1.6	9.7E−26
C4B63_23g130	Fumarate Hydratase Class I, Cytosolic	1.6	1.3E−19
C4B63_34g287	Glycerate Kinase^a^	3.5	8.7E−06
C4B63_9g273	Phosphoenolpyruvate Carboxykinase [ATP], Glycosomal	1.7	1.2E−18
C4B63_11g74	Pyruvate Dehydrogenase E1 Beta Subunit	1.8	4.6E−40
C4B63_9g396	Sedoheptulose-1,7-Bisphosphatase	1.7	2.4E−21
C4B63_57g98	Succinyl-CoA Ligase	1.7	1.5E−29
C4B63_11g8	Succinyl-CoA Synthetase Alpha Subunit	2.8	5.8E−85
C4B63_28g114	Triosephosphate Isomerase	1.7	2.5E−28
C4B63_63g46	2,3-Bisphosphoglycerate-Independent Phosphoglycerate Mutase	1.7	2.0E−31
C4B63_18g232	Glyceraldehyde 3-Phosphate Dehydrogenase	2	6.5E−35
*Lipid biosynthetic process*			
C4B63_18g204	3-Hydroxy-3-Methylglutaryl-CoA Reductase	1.8	3.9E−49
C4B63_32g225	3-Hydroxy-3-Methylglutaryl-CoA Synthase	2.2	2.5E−71
C4B63_127g51	Acetyl-CoA Carboxylase	1.5	2.7E−15
C4B63_12g385	Acyl Carrier Protein, Mitochondrial	1.5	1.4E−16
C4B63_8g510	Acyl-ACP Thioesterase	1.5	5.4E−15
C4B63_24g298	CDP-Diacylglycerol–Inositol 3-Phosphatidyltransferase^a^	1.9	1.7E−07
C4B63_300g18	Glycerophosphoryl Diester Phosphodiesterase Family	1.5	2.6E−03
C4B63_127g56	Inositol Polyphosphate 1-Phosphatase^a^	2.1	1.3E−22
C4B63_44g211	Mevalonate Kinase	1.6	2.9E−27
C4B63_227g9	Mevalonate-Diphosphate Decarboxylase	1.5	1.2E−11
*Carboxylic acid metabolic process*			
C4B63_2g509	Arginyl-tRNA Synthetase^a^	1.6	3.1E−22
C4B63_7g79	Aspartyl-tRNA Synthetase^a^	1.5	1.3E−22
C4B63_21g261	Isocitrate Dehydrogenase [NADP], Mitochondrial Precursor	1.5	5.6E−15
C4B63_12g221	Pyruvate Phosphate Dikinase^a^	1.9	2.5E−32
C4B63_18g212	Valyl-tRNA Synthetase	1.5	2.3E−24
*Aspartate family amino acid metabolic process*			
C4B63_32g214	2-Amino-3-Ketobutyrate Coenzyme A Ligase^a^	2.6	2.5E−51
C4B63_8g483	Asparagine Synthetase A	2.1	1.9E−70
C4B63_28g64	Aspartate Aminotransferase	1.5	5.3E−12
*Cellular nitrogen compound biosynthetic process*			
C4B63_5g418	DNA-Directed RNA Polymerase III Subunit	1.6	1.5E−04
C4B63_25g113	GMP Reductase	1.5	1.8E−19
C4B63_157g32	Inosine-5'-Monophosphate Dehydrogenase^a^	4.1	2.3E−11
C4B63_5g795	O-Phosphoseryl-tRNA(Sec) Selenium Transferase	2.2	2.0E−09
C4B63_35g130	Orotidine-5-Phosphate Decarboxylase/Orotate Phosphoribosyltransferase^a^	1.5	6.5E−10
C4B63_42g103	Pyridoxal Kinase	2	2.8E−22
*Peptidyl-amino acid modification*			
C4B63_40g25	Biotin-Acetyl-CoA-Carboxylase Ligase	1.5	3.8E−20
C4B63_225g41	Cyclophilin^a^	3	1.8E−03
C4B63_47g52	Deoxyhypusine Synthase^a^	1.9	7.5E−19
*Purine nucleotide metabolic process*			
C4B63_8g559	Adenine Phosphoribosyltransferase	1.6	6.1E−24
C4B63_91g38	ATP Synthase F1, Alpha Subunit	1.9	3.1E−04
C4B63_12g330	ATP Synthase Subunit Beta, Mitochondrial	1.7	3.0E−30
C4B63_47g72	Enolase	2.9	2.9E−65
C4B63_13g270	Fructose-Bisphosphate Aldolase, Glycosomal	1.5	2.1E−08
C4B63_30g161	Glutamine Synthetase^a^	1.6	2.7E−21
C4B63_10g404	Guanine Deaminase	2.4	5.5E−26
C4B63_102g44	Hypoxanthine–Guanine Phosphoribosyltransferase	1.6	1.9E−07
C4B63_56g86	Methylthioadenosine Phosphorylase	1.9	1.1E−35
C4B63_18g164	S-Adenosylmethionine Synthetase	2.6	1.3E−96
*Transmembrane transport*			
C4B63_34g105	Adenosine 3'-Phospho 5'-Phosphosulfate Transporter 2	1.5	4.3E−15
C4B63_41g1061c	ATPSynthase Subunit	1.8	3.7E−03
C4B63_8g2697c	ATPase Subunit 9	1.7	5.0E−20
C4B63_6g236	Cation Transporter^a^	2.6	4.6E−89
C4B63_19g205	Glycosomal Transporter (GAT3)	1.5	2.2E−20
C4B63_85g60	Tricarboxylate Carrier	2.5	9.2E−72
*Protein transport, intracellular transport, vesicle-mediated transport*			
C4B63_12g295	GTP-Binding Nuclear Protein Rtb2	1.7	3.4E−32
C4B63_46g30	Ran-Binding Protein 1	1.7	5.8E−17
C4B63_184g36	Vesicle-Associated Membrane Protein 7	1.6	4.6E−03
*Microtubule-based movement, signal transduction, protein homooligomerisation*			
C4B63_27g173	Kinesin^a^	1.6	2.3E−22
C4B63_150g11	Mucin-Associated Surface Protein (MASP)	8.2	2.8E−17
C4B63_157g35	Potassium Voltage-Gated Channel	2.4	9.8E−04
*Cell division, nucleosome assembly, DNA replication, DNA repair*			
C4B63_20g747c	Cyclin-Dependent Kinases Regulatory Subunit	1.5	6.9E−10
C4B63_31g223	Meiotic Recombination Protein SPO11	1.5	1.1E−11
C4B63_295g15	Nucleosome Assembly Protein-Like Protein^a^	2.2	7.5E−27
C4B63_9g314	DNA Replication Licensing Factor MCM3/4^a^	1.5	7.4E−11
C4B63_26g205	Proliferative Cell Nuclear Antigen (PCNA)	1.7	5.7E−31
C4B63_21g281	DNA Repair and Recombination Helicase Protein PIF5	1.6	2.2E−27
C4B63_27g165	Endonuclease III	1.5	1.4E−09
C4B63_2g740	Nucleic Acid-Binding, Ob-Fold	1.5	7.6E−14
C4B63_24g173	O-6 Methyl-Guanine Alkyl Transferase^a^	2.5	6.2E−10
*RNA modification, rRNA processing, mRNA transport*			
C4B63_43g125	Centromere/Microtubule Binding Protein Cbf5	1.6	3.1E−30
C4B63_87g6	Isy1 Splicing Protein-Like Protein	1.5	8.7E−16
C4B63_6g104	Mitochondrial Rna Binding Protein	1.8	2.1E−27
C4B63_2g1123c	Small Nuclear Ribonucleoprotein Sm-F	1.8	9.1E−15
C4B63_56g50	Surp Module (Swap Domain)	1.5	1.7E−09
C4B63_207g2	Fibrillarin^a^	1.9	1.4E−17
C4B63_34g118	H/Aca Ribonucleoprotein Complex Non-Core Subunit Naf1	1.9	7.9E−31
C4B63_46g78	Nucleobase Transporter	1.5	1.1E−06
C4B63_44g246	Nucleolar Protein	1.6	1.7E−23
C4B63_34g308	Ribosomal RNA Methyltransferase	1.7	1.3E−23
C4B63_7g263	Ribosomal RNA Processing Protein 6	1.5	8.5E−16
C4B63_163g43	Enhancer Of Yellow 2 Transcription Factor^a^	1.6	7.9E−03
C4B63_19g26	Guide RNA-Binding Protein Of 21 KDa	1.6	3.9E−25
*Translation*			
C4B63_58g90	Elongation Factor 1-Alpha^a^	2.1	1.3E−02
C4B63_5g793	Elongation Factor 1-Gamma (EF-1-Gamma)^a^	4.1	1.9E−05
C4B63_13g192	Elongation Factor G1, Mitochondrial	1.7	9.7E−31
C4B63_35g189	Eukaryotic Translation Initiation Factor 1A	1.8	6.9E−27
C4B63_19g167	Eukaryotic Translation Initiation Factor 5A^a^	2.7	1.1E−07
C4B63_19g181	Nucleotide-Binding Alpha–Beta Plait Domain Superfamily	1.6	4.7E−10
C4B63_275g8	Polyubiquitin	3.5	4.2E−13
C4B63_304g8	40S Ribosomal Protein S13^a^A	2.1	5.1E−07
C4B63_76g132	50S Ribosomal Protein L22	1.6	2.4E−05
C4B63_30g166	60S Ribosomal Protein L19^a^A	4	5.9E−05
C4B63_6g1208c	Ribosomal Protein S29^a^A	2.8	4.9E−05
C4B63_20g319	Translation Elongation Factor 1-Beta 25 kDa^a^	2.5	1.5E−90
C4B63_281g8	Translation Initiation Factor eIF2B Delta Subunit	1.5	3.6E−04
C4B63_13g195	Ubiquitin/Ribosomal Protein S27A	1.8	5.0E−29

See Additional file 3: Table S2 for complete data

^a^Transcripts with > 1 copy in the corresponding Gene Ontology category. Those ribosomal RNA transcripts in which the superscript 'a' is followed with an uppercase 'A' are those with > 1 type of the same group

Of the 752 (41.3%) downregulated transcripts in the BZ-resistant *T. cruzi* line, including those with functional annotation and hypothetical proteins, 263 (34.9%) presented GO terms on biological processes (251 enriched and 12 without enrichment), 130 (17.3%) transcripts did not present GO terms on biological processes and 359 (47.7%) transcripts with functional annotation and hypothetical proteins had no GO-associated term.

According to the GO enrichment analysis, a group of ribosomal proteins related to translation was downregulated in the DE dataset with copies (marked with superscript 'a' in Tables 2 and 3) and different types (marked with uppercase 'A' in Table 3).

Three GO terms associated with protein modification were identified in the downregulated transcript analysis: proteolysis, protein phosphorylation and protein folding. Twelve transcripts, such as ATP-dependent zinc metallopeptidase (up to 3.7-fold downregulated), proteasome beta 7 subunit (1.6-fold downregulated) and ubiquitin carboxyl-terminal hydrolase (1.6-fold downregulated) were included in the proteolysis category. Seven transcripts were included in the GO term protein phosphorylation, including protein kinase (up to 4.6-fold downregulated), protein tyrosine phosphatase-like protein (1.8-fold downregulated), inorganic pyrophosphatase (1.7-fold downregulated) and CDC2-related kinase 12 (1.7-fold downregulated). The protein folding category contained several proteins responsible for maintenance of the structural stability of peptides, including chaperone DNAJ protein (1.6-fold downregulated), chaperonins (2.1- to 2.2-fold downregulated), heat shock proteins 70, 10 kDa, DNAJ (1.5- to 2.6-fold downregulated) and T-complex proteins (1.5- to 1.6-fold downregulated).

The GO term oxidation–reduction process contained transcripts involved in the oxidative stress response, such as superoxide dismutase (2-fold downregulated), thioredoxin-like (2.5-fold downregulated), glutaredoxin (2.2-fold downregulated), peroxisomal biogenesis factor 11 (2.1-fold downregulated), glycerol-3-phosphate dehydrogenase, NAD-dependent N-terminal (2.2-fold downregulated) and peroxiredoxin (up to 1.7-fold downregulated). The oxidation–reduction category is obsolete in the current version of GO, where it is considered a molecular function and not a biological process. However, the GAF file used for enrichment maintained this GO and is a process of great relevance for the investigated BZ resistance phenotype.

Twelve transcripts downregulated in the BZ-resistant *T. cruzi* lines belonged to two GO terms associated with energy metabolism: generation of precursor metabolites and energy (10 transcripts) and glycolytic process (2 transcripts). These two terms are closely related, and the transcripts identified in the glycolytic process are glyceraldehyde 3-phosphate dehydrogenase (2-fold downregulated) and 2,3-bisphosphoglycerate-independent phosphoglycerate mutase (1.7-fold downregulated). The GO categories included glycerate kinase (up to 3.5-fold downregulated), succinyl-CoA synthetase alpha subunit (2.8-fold downregulated) and sedoheptulose-1,7-bisphosphatase (1.7-fold downregulated), among others.

Two GO terms associated with lipid metabolism were identified in the downregulated transcript functional enrichment analysis: the lipid biosynthetic process and carboxylic acid metabolic process. Some of the terms for lipid biosynthetic process include transcripts such as mevalonate-kinase (1.6-fold downregulated), mevalonate-diphosphate decarboxylase (1.5-fold downregulated), acetyl-COA carboxylase (1.5-fold downregulated) and 3-hydroxy-3-methylglutaryl-CoA synthase (2.2-fold downregulated). Five transcripts were included in the carboxylic acid metabolic process: arginyl-tRNA synthetase (1.6-fold downregulated), aspartyl-tRNA synthetase (1.5-fold downregulated), valyl-tRNA synthetase (1.5-fold downregulated), pyruvate phosphate dikinase (1.9-fold downregulated), isocitrate dehydrogenase (NADP) and mitochondrial precursor (1.5-fold downregulated). The enzymes involved in the mevalonate pathway have been found to be upregulated in early *T. cruzi* amastigotes during host cell infection, suggesting that the parasite generates sterols and fatty acids to support replication and membrane homeostasis [14]. In the cellular nitrogen compound biosynthetic process, six transcripts were downregulated in the BZ-resistant *T. cruzi* line, including inosine-5′-monophosphate dehydrogenase (4.1-fold downregulated), pyridoxal kinase (2-fold downregulated),* O*-phosphoseryl-tRNA(sec) selenium transferase (2.2-fold downregulated) and GMP reductase (1.5-fold downregulated).

Eleven transcripts belonging to the purine nucleotide metabolic process were downregulated in the BZ-resistant *T. cruzi* line. Some transcripts included in this group are ATP synthase subunits (1.7- to 1.9-fold downregulated), guanine deaminase (2.4-fold downregulated) and* S*-adenosylmethionine synthetase (2.6-fold downregulated). The GO term peptidyl-amino acid modification was identified with three associated transcripts: biotin-acetyl-coA-carboxylase ligase (1.5-fold downregulated), cyclophilin (up to 3-fold downregulated) and deoxyhypusine synthase (1.9-fold downregulated). Another GO term with three associated transcripts was the aspartate family amino acid metabolic process, which included 2-amino-3-ketobutyrate coenzyme A ligase (2.6-fold downregulated), asparagine synthetase A (2.1-fold downregulated) and aspartate aminotransferase (1.5-fold downregulated).

Three GO terms were enriched with one transcript each: protein homooligomerisation, signal transduction and microtubule-based movement with potassium voltage-gated channel (2.4-fold downregulated), MASP (8.2-fold downregulated) and kinesin (1.6-fold downregulated), respectively. Four GO terms, namely transmembrane transport, vesicle-mediated transport, intracellular transport and protein transport, were identified, with nine transcripts belonging to these terms, including RAN-binding protein 1 (1.7-fold downregulated), GTP-binding nuclear protein Rtb2 (1.7-fold downregulated), vesicle-associated membrane protein 7 (1.6-fold downregulated), glycosomal ABC transporter (GAT3) (1.5-fold downregulated) and other carriers.

Seventy-one transcripts encoding proteins associated with translation (GO: 0006412), including different types of 40S and 60S ribosomal proteins, ubiquitin-associated proteins and elongation factors, were 1.5- to 4.1-fold downregulated in the BZ-resistant *T. cruzi* population (Table 3). GO terms associated with RNA modification, ribosomal RNA (rRNA) processing and messenger RNA (mRNA) transport were enriched and contained 13 different transcripts (Table 3), including nucleobase transporter (1.5-fold downregulated), small nuclear ribonucleoprotein Sm-F (1.8-fold downregulated) and other transcripts that were 1.5- to 1.9-fold downregulated in the BZ-resistant population. Four GO terms were functionally related: cell division, nucleosome assembly, DNA replication and DNA repair. These terms included 11 transcripts, such as DNA repair and recombination helicase protein Pif5 (1.6-fold downregulated), *O*-6 methyl-guanine alkyl transferase (2.5-fold downregulated), DNA replication licencing factors (1.5- to 1.7-fold downregulated), nucleosome assembly protein (1.7- to 2.2-fold downregulated) and meiotic recombination protein Spo11 (1.5-fold downregulated).

**Fig. 4:**
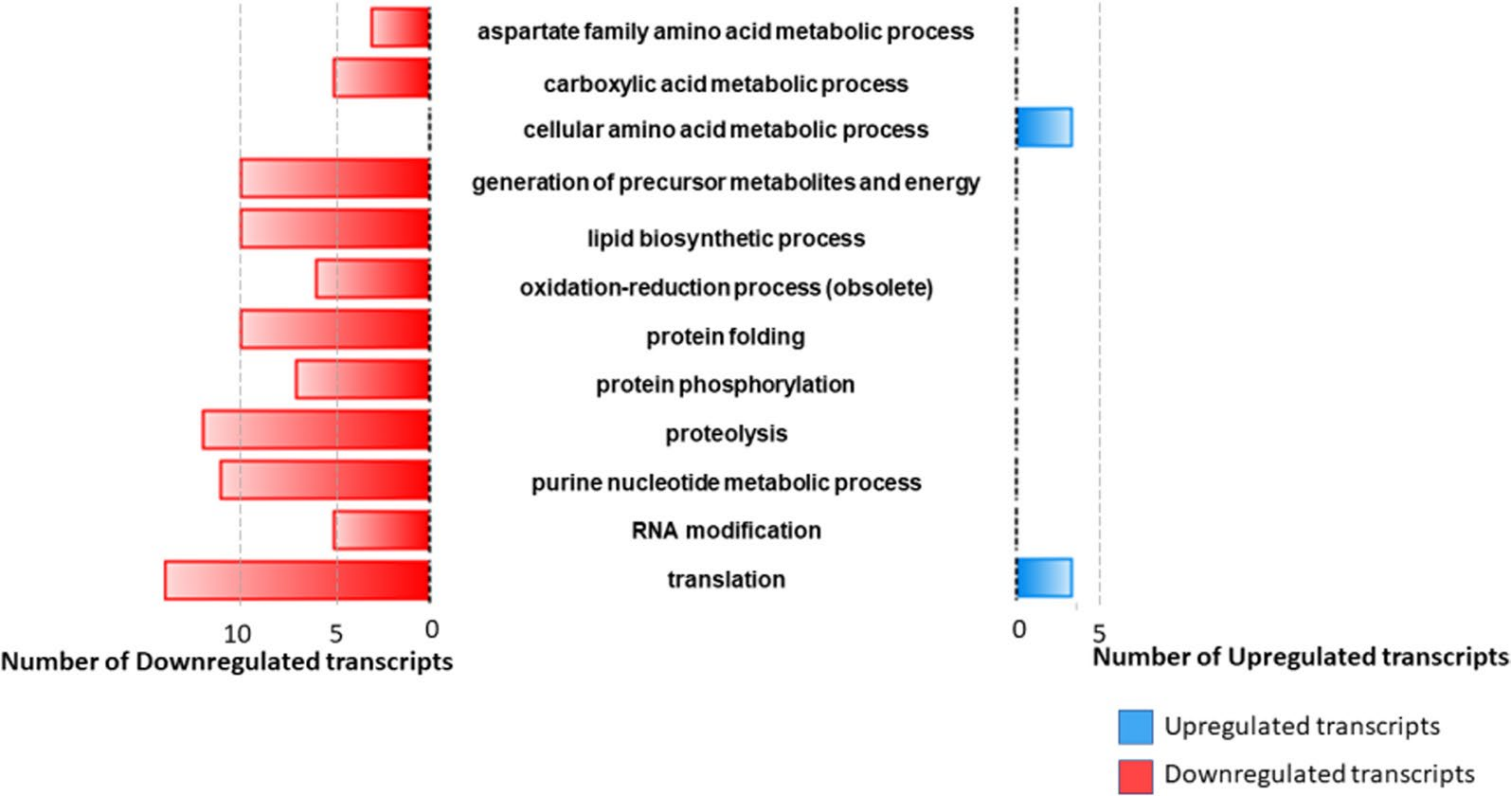
Differentially expressed transcripts for the most representative Gene Ontology (GO)-enriched terms for the biological process category. The most representative GO terms for the enriched dataset. Blue bars represent the total number of upregulated transcripts for each term, and red bars represent the total number of downregulated transcripts for each category

The enrichment analysis of the downregulated transcripts identified several altered biological processes. The main GO terms can be grouped into post-transcriptional modifications, energy metabolism and amino acid metabolism. Despite the different ontologies, related biological processes can be observed in the categories identified among the DE transcripts. A summary of the functional enrichment analysis results is presented in Fig. 4.

More functional categories were identified as enriched; however, due to the GO-structured hierarchy and uninformative basal categories, we focused our analysis on the more derived biological processes. The complete biological process-enriched data are shown in Additional file 3: Table S2.

### Transcripts without GO enrichment for biological processes

Some important DE transcripts did not show functional enrichment; however, they were important for the BZ resistance phenotype in the parasite. These DE transcripts had associated GO terms but were not enriched for any category (Additional file 4: Table S3). Some of the identified transcripts upregulated in the BZ-resistant population without GO enrichment are hexose transporters (1.5- to 2.4-fold upregulated), multidrug resistance protein E (1.9-fold upregulated), ABC transporters (2.2- to 2.9-fold upregulated), dual specificity protein phosphatase (1.5- to 1.8-fold upregulated), ascorbate peroxidase (2.5-fold upregulated), arginase (1.7-fold upregulated), superoxide dismutase (1.6-fold upregulated), thioredoxin (1.5-fold upregulated) and adenine phosphoribosyl transferase (1.5-fold upregulated).

**Fig. 5:**
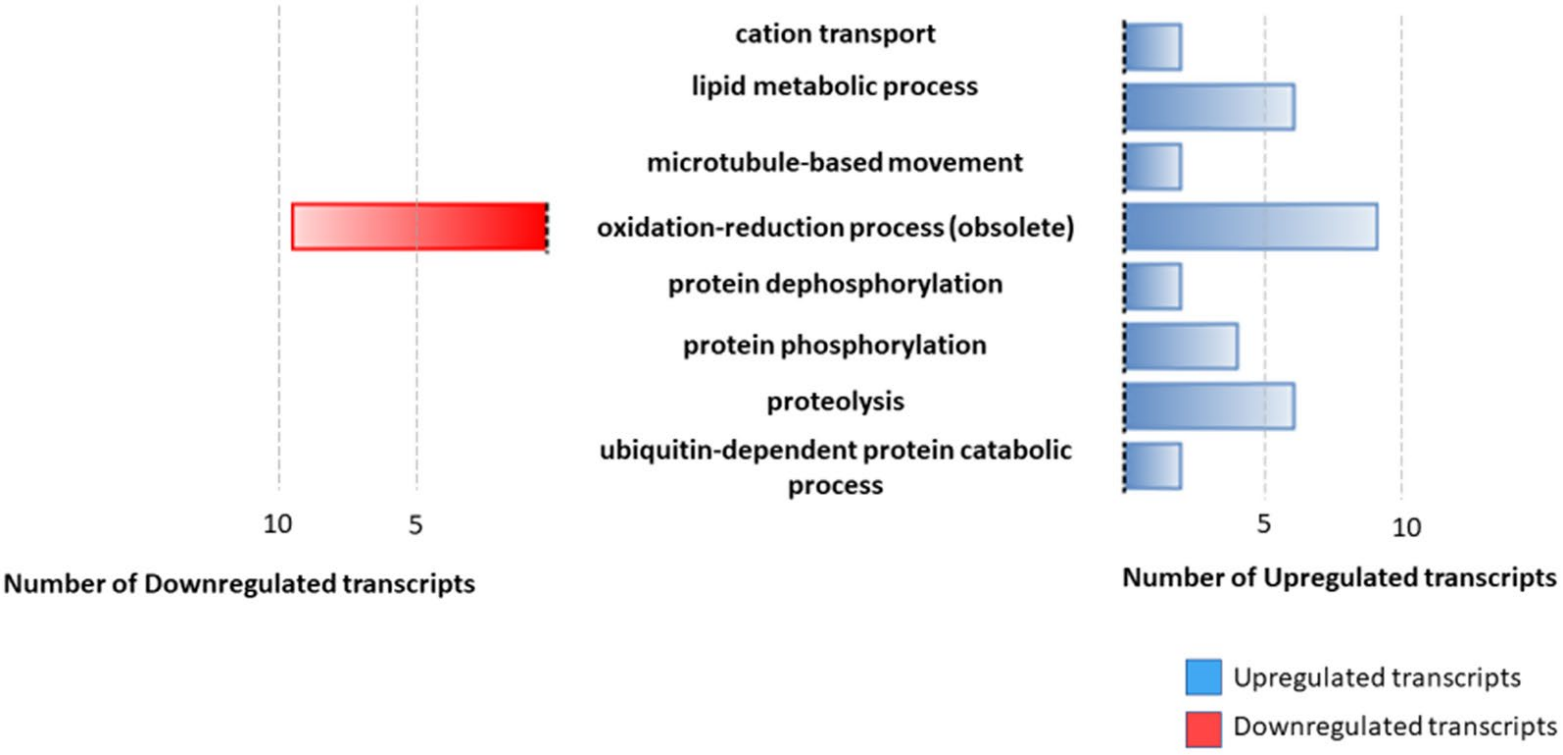
Differentially expressed transcripts for the most representative Gene Ontology (GO) terms not enriched for the biological process category. The figure shows the most representative GO terms for the not enriched dataset. Blue bars represent the total number of upregulated transcripts for each term, and red bars represent the total number of downregulated transcripts for each category

Among the downregulated transcripts, we identified two copies of prostaglandin F synthase (1.7-fold downregulated), phosphomevalonate kinase protein (1.8-fold downregulated), malic enzyme (3.6-fold downregulated), oxoglutarate/iron-dependent dioxygenase (1.8-fold downregulated) and ABC transporter (1.5-fold downregulated). The main categories identified through detailed GO analysis are shown in Fig. 5.

### Transcripts without associated GO term

Some transcripts were found to have an associated functional annotation but no related GO term for biological processes, such as transcripts with predicted function and transcripts without predicted function (annotated as hypothetical or unknown function) (Additional file 5: Table S4). A total of 1085 transcripts with predicted functions were not associated with any GO term (727 upregulated and 358 downregulated). The group of transcripts with predicted functions but without any associated GO terms include several members of multigene families, such as mucins, MASPs, retrotransposon hot spot proteins (RHS) and trans-sialidases. Other relevant transcripts also appeared in this group, such as DNAJ chaperone protein, ABC transporters and RNA-binding proteins.

A group of transcripts identified without a predicted function was annotated as hypothetical or unknown function. The level of detail of functional annotation may limit the interpretation of the metabolic pathways in which these transcripts are involved. This lack of knowledge is represented by the absence of an associated GO term for the identified DE transcripts. In total, 297 DE transcripts without a predicted function were identified (166 upregulated and 131 downregulated). Additional file 6: Table S5 contains transcripts with unknown functions and without GO term associations.

## Discussion

Chagas disease remains a serious public health problem that lacks efficient and well-tolerated alternative treatments. Only two medicines are currently available for clinical treatment: BZ and NFX; however, both drugs present side effects and have low curative efficacy, mainly during the chronic phase of the disease. Long-term treatment protocols and the occurrence of drug-resistant *T. cruzi* strains are other limitations of CD treatment [3]. Thus, new therapeutic agents and novel targets for drug development are urgently required. In the present study, we performed a comparative transcriptomic analysis of wild-type and BZ-resistant *T. cruzi* populations using high-throughput RNAseq to enhance our understanding of metabolic pathways related to clinical drug resistance and identify promising molecular targets for developing new drugs to treat CD.

Massive RNAseq of *T. cruzi* is a challenge because of the number of repetitive sequences present in the parasite genome and the difficulty in obtaining high-quality reference genomes. The repetitive nature of the *T. cruzi* genome makes it difficult to quantify gene expression and accurately determine whether certain genes are upregulated or belong to regions with multiple copies. The genome of *T. cruzi* is biologically diverse and complex. Our analysis was based on the most recent haploid assembly of related *T. cruzi* strain Dm28c, which identified 74 multigene families and approximately 5000 single-copy genes, of which about 1400 had functional annotation [22].

One example of the challenge facing researchers in this context is the transcript “UDP-Gal” or “UDP-GLCNAc-dependent glycosyltransferase”, an enzyme related to initial steps of mucin glycosylation [30]. This transcript appeared to be 180-fold upregulated in the BZ-resistant *T. cruzi* population, but it was not considered to be DE due to the associated high FC. The gene for this enzyme is located on chromosome 31 of *T. cruzi*, which has extra copies in all *T. cruzi* discrete typing units (DTUs) and a high concentration of genes associated with energy metabolism [31]. The genes encoding surface proteins, such as “trans-sialidases”, “mucins”, “MASP)” and “surface protease GP63”, are also part of this challenge, since that they are the largest multigene families present in the *T. cruzi* genome. These transcripts are responsible for the composition of the parasite surface membrane and are crucial for protection against host immune response; a relationship exists between the diversity of these genes in the genome and the number of potential hosts [32, 33]. In our study, the surface proteins trans-sialidases and GP-63 were found to be upregulated by two- to 30-fold and by 1.6- to 6.8-fold, respectively, in the BZ-resistant *T. cruzi* populations. The high transcriptomic variability observed in this study could be influenced by many factors, including a complex genomic organisation, a limited quality sequencing data, a large number of multigene families and potential aneuploidy in response to stress and drug pressure [31, 33].

Owing to the difficulties present in the assembly of the genomes and the genetic complexity of *T. cruzi*, information on the genes present in the databases is limited. Functional categories are represented from the most generic to the most specific ontology in a tree structure so that the same transcript can be associated with > 1 GO term. Furthermore, the presence of large multigene families in the *T. cruzi* genome adds a layer of redundancy to the analysis. In the present study, the analysis focused on the identified biological processes, since this functional category describes biological mechanisms that may be used to infer parasite responses to selective pressure.

Antioxidant defense enzymes act in concert to protect parasites against different reactive oxygen species produced by cellular metabolism and external agents, including products of the host immune response and drug metabolism. This mechanism is associated with the GO term oxidation–reduction process (GO: 0055114) identified in the enrichment analysis. Several other mechanisms involved in antioxidant defense have previously been documented to show association with BZ resistance in *T. cruzi* [34]. We found that the superoxide dismutase (SOD) transcript, which is a part of this metabolic pathway, is twofold downregulated in BZ-resistant *T. cruzi* population (Additional file 3: Table S2). Another transcript annotated as SOD appeared to be 1.6-fold upregulated in the same population (Additional file 3: Table S2). SOD protects the parasite against superoxide radicals, which are converted to oxygen and hydrogen peroxide [35, 36]. Fe-SOD has four distinct isoforms that act in different cellular compartments and are part of a superfamily [37]. Previous studies have shown increased mRNA Fe-SOD-A levels, protein expression and Fe-SOD enzyme activity in a *T. cruzi* population with in vitro-induced resistance to BZ (17 LER) [11]. APX, another enzyme involved in antioxidant defense, showed 2.5-fold increased expression in the BZ-resistant *T. cruzi* population (Additional file 3: Table S2). APXs are class I haem-containing enzymes that catalyse H_2_O_2_-dependent oxidation of ascorbate to water [38]. Consistent with our results, Nogueira et al. [13] observed that APX levels were enhanced in *T. cruzi* populations with in vitro*-*induced (17 LER) and in vivo-selected (BZR) resistance to BZ. Glutaredoxin and thioredoxin-like transcripts are also involved in the oxidation–reduction process. Glutathione scavenges reactive nitrogen and oxygen species, contributing to redox homeostasis. The thioredoxin system is responsible for selenium metabolism and acts as a hydrogen donor for the enzyme glutathione peroxidase [39].

One of the main resistance mechanisms developed by *T. cruzi* has been linked to the inhibition of the bioactivation of BZ and NFX by nitroreductases [9, 10]. In the present study, one transcript encoding nitroreductase (ID: C4B63_56g60) was 1.8-fold downregulated in the BZ-resistant *T. cruzi* population. These data are supported by previous findings showing the downregulation of NTR transcript levels in *T. cruzi* lines resistant to nitroheterocyclic drugs [9, 10]. In agreement with previously reported microarray [40] and proteome data [41], prostaglandin F synthase (PGFS), an enzyme that has been correlated with *T. cruzi* resistance to BZ, was also identified in our study as being 1.7-fold downregulated in the BZ-resistant *T. cruzi* population. Recently, Santi et al. [42] demonstrated that deletion of the *PGFS* gene in *T. cruzi* did not alter the parasite’s resistance to BZ or NFX, but it did reduce tolerance to oxidative stress and infectivity. These authors suggested that PGFS plays a regulatory role in defense against oxidative stress and parasite infectivity.

The category protein folding contains chaperone DNAJ protein (1.6-fold downregulated), chaperonins (2.1- to 2.2-fold downregulated), 70- and 10-kDa heat shock proteins, DNAJ (1.5- to 2.6-fold downregulated) and T-complex proteins (1.5- to 1.6-fold downregulated). These transcripts are associated with the stress response and may be responsible for correct protein folding or reductase or oxidase activity, as described previously for DNAJ chaperone [43]. The GO term protein phosphorylation is an important post-transcriptional modification that contains several enriched transcripts associated with a protein kinase (up to 4.6-fold downregulated), protein tyrosine phosphatase-like protein (1.8-fold downregulated), inorganic pyrophosphatase (1.7-fold downregulated) and cdc2-related kinase 12 (1.7-fold downregulated). Despite the small number of characterised *T. cruzi* phosphatases, the role of tyrosine phosphatases is important in several biological functions, such as cell cycle and immune response. These enzymes are divided into four distinct classes based on their catalytic domains and active substrate [44]. One of these four categories, proteolysis, includes transcripts such as ATP-dependent zinc metallopeptidase (up to 3.7-fold downregulated), proteasome beta 7 subunit (1.6-fold downregulated) and ubiquitin carboxyl-terminal hydrolase (1.6-fold downregulated). These proteins are related to protein degradation and the ubiquitin pathway; their half-life is associated with cellular function and is regulated by the parasite. Ubiquitin metabolism has been identified in other trypanosomatids, and previously published data suggest that alterations in protein ubiquitination may contribute to the degradation of oxidised proteins, thereby protecting the parasite against oxidative stress [45, 46].

Protein transport, transmembrane transport, intracellular transport, vesicle-mediated transport and microtubule-based movement were GO terms associated with molecule transport. Several carriers were identified, including vesicle-associated membrane protein 7 (1.6-fold downregulated), GAT3 (1.5-fold downregulated) and adenosine 3′-phospho 5′-phosphosulfate transporter 2 (1.5-fold downregulated). GAT3 is related to glucose uptake from the cytosol to the glycosomal lumen, and was previously identified to be upregulated in the antimony-resistant *Leishmania infantum* line [47, 48]. GAT3 has been hypothesised to act as a drug efflux pump in its role in the resistance phenotype, preventing it from acting on parasites. In the present study, several ABC transporter gene transcripts were 1.8- to 2.9-fold upregulated and 1.5-fold downregulated in the BZ-resistant *T. cruzi* population (Additional file 3: Table S2). These transporters have a strong association with the drug-resistance phenotype, since they work as drug efflux pumps to protect the parasite from the action of these molecules [49, 50]. The hexose transporters (HT) transcripts are membrane proteins involved in the uptake of glucose and other hexoses into the cell [51]. In our study, the transcript encoding HT showed 1.5- to 2.4-fold increased expression in the BZ-resistant *T. cruzi* population. In a previous study, the transcript level of the HT gene was observed to be more highly expressed in the BZ-resistant *T. cruzi* population. However, the authors observed a 40% reduction in transporter activity in this population, suggesting the existence of a regulatory mechanism at the protein activity level [52].

The GO terms purine nucleotide metabolic process, peptidyl-amino acid modification and cellular nitrogen compound biosynthetic process were identified and were related to amino acid metabolism. Transcripts associated with amino acid metabolism have already been characterised, and a relationship with BZ resistance in *T. cruzi* has been established [19, 53]. Adenine phosphoribosyltransferase (APRT) is a protein that participates in the purine recycling pathway in trypanosomatids by capturing purines from the host and synthesising nucleotides. In our study, the APRT transcript was 1.6-fold downregulated in the BZ-resistant *T. cruzi* population. Our findings are corroborated by those of a previous transcriptome study, which showed that the APRT transcript was 5.2-fold downregulated in the population of *T. cruzi* that is naturally resistant to BZ [19]. The authors of that study overexpressed the APRT transcript in susceptible and resistant parasites, both of which became more susceptible to BZ, confirming an association of APRT with the BZ-resistant phenotype. Another identified transcript, tyrosine aminotransferase (TAT), is a key enzyme in a number of metabolic pathways, including the biosynthesis and degradation of amino acids and carbohydrate metabolism [53]. In our study, TAT transcript copies were 1.7-, 1.9- and threefold upregulated in the BZ-resistant *T. cruzi* populations. In agreement with our data, proteomic analysis also showed that this enzyme was overexpressed in BZ-resistant *T. cruzi* strains [41]. The GO terms nucleosome assembly, DNA replication and DNA repair, were associated with cell cycle, DNA replication and repair, respectively, and were identified as enriched. Several transcripts were identified, including the DNA repair and recombination helicase protein Pif5 (1.6-fold downregulated),* O*-6 methyl-guanine alkyl transferase (2.5-fold downregulated), DNA replication licencing factors (1.5- to 1.7-fold downregulated), nucleosome assembly protein (1.7- to 2.2-fold downregulated) and meiotic recombination protein Spo11 (1.5-fold downregulated). GO nucleosome assembly is associated with the structural aspect of chromatin, which could be an expression of regulation that facilitates or hinders the access of polymerases to certain sites in the genome [54]. The transcripts identified for the GO term DNA repair participate in protecting the parasite genome against damage from oxidative stress. Recent findings suggest that these transcripts play an important role in signalling damage caused by oxidative stress and are a promising target for CD chemotherapy [55].

GO terms associated with RNA modification, rRNA processing and mRNA transport were enriched and contained several transcripts, including the nucleobase transporter (1.5-fold downregulated) and small nuclear ribonucleoprotein Sm-F (1.8-fold downregulated). The biological processes associated with the identified terms may be related to post-transcriptional modifications and are important for maintaining RNA stability in RNA-binding proteins (RBPs). The GO term translation is associated with transcripts already characterised as eukaryotic translation initiation factor 5A (eIF5A), which plays an essential role in the elongation of the amino acid chain during translation. In our study, this transcript had two copies that were 2.7- and 1.6-fold downregulated in the BZ-resistant *T. cruzi* population. Previous studies have also shown that eIF5A protein levels were twofold lower in BZ-resistant *T. cruzi* populations. In addition, mutant parasites that overexpress eIF5A are three to five times more susceptible to BZ [56]. Among the categories enriched in the upregulated transcripts in the BZ-resistant *T. cruzi* population, translation, aromatic amino acid metabolism and arginine metabolic processes were observed among the categories enriched in the upregulated transcripts in the BZ-resistant *T. cruzi* population. Results from earlier studies suggest an important role for arginine in the metabolism of *T. cruzi*, given the high specificity of its transporter in the parasite. The metabolism of this amino acid is related to the energy reserve metabolism of the parasite and an increase in the enzyme arginine kinase under conditions of oxidative stress and chemotherapy [57, 58]. Changes in glutamine biosynthesis metabolism may underlie the response to the effect of BZ, such as changes in this pathway due to the presence of ammonium and azole compounds, as has been already described [59, 60]. In the same group of transcripts, the processes of RNA metabolism and translation were enriched, which may suggest either a high tolerance of these processes to changes or a lower impact on the selective pressures of the drug. Among the downregulated transcripts, several biological processes were identified as being enriched. Amidst the diversity of the functions identified and their positions in the GO tree, the GO terms identified were translation, protein phosphorylation, nucleosome organisation, proteolysis, DNA repair, purine metabolism and RNA modification.

The transcript of the gene encoding the enzyme ADH was 2.8-fold upregulated in the BZ-resistant *T. cruzi* population. ADHs are a class of oxidoreductases that catalyse the reversible oxidation of ethanol to acetaldehyde. González et al. [61] suggested that overexpression of this enzyme counteracts mitochondrial and cell membrane damage after treatment with BZ. In the same study, these authors also observed that this enzyme is fourfold more highly expressed in the naturally BZ-resistant *T. cruzi* population and that mutant parasites which overexpressed this enzyme showed greater resistance to BZ and glyoxal, reduced damage to cell and mitochondrial membranes and decreased concentration of reactive oxygen species [61].

CyP, a peptidyl-prolyl cis/trans isomerase, is a key molecule with diverse biological functions that include roles in molecular folding, stress response, immune modulation and signal transduction. In the present study, seven CyP transcripts were found to be 1.5- to threefold downregulated in the BZ-resistant *T. cruzi* population. Proteomic analysis showed that the TcCyP19 isoform is more abundant in the BZ-resistant *T. cruzi* population [41]. Molecular characterisation studies revealed a twofold increase in mRNA and TcCyP19 protein levels in populations of *T. cruzi* with in vitro-induced and selected in vivo resistance to BZ [62]. Cyclophilins are a large multigene family containing various isoforms; thus, other transcripts that encode these proteins can replace the function of TcCyP19.

Based on the enriched functional categories found and the expression of transcripts documented in the literature, the *T. cruzi* resistance phenotype to BZ is strongly influenced by the fight against oxidative stress [63, 64]. The high tolerance of the parasite to mutations allows major changes in the regulatory mechanisms of gene expression and results in a high diversity of molecular mechanisms that can be selected under high selective pressure [33]. The combating of reactive oxygen species is crucial in *Trypanosoma cruzi* for the establishment of the infection, although the mechanism underlying the action of BZ is yet to be completely elucidated. Low-molecular-weight thiols produced by BZ exert a high impact on the parasite [8]. According to these results, amino acid metabolism, DNA repair and energy metabolism have a strong impact on oxidative stress and may be an alternative method for the parasite to recycle metabolites that are potentially harmful to the cell [55, 57].

The change in aminoacylation of tRNA-arginyl is associated with the processing of amino acids, as previously mentioned (as one of the upregulated transcripts). The biological processes of nucleosome alteration, translation, and mRNA export are related to post-transcriptional steps of gene expression. This concentration of negatively selected biological processes at this level may indicate a higher impact of the processes that control gene expression in the resistance phenotype, suggesting a possible relevant aspect in earlier stages (at the genomic level) or later stages (post-transcriptional processes).

Data reported in the literature show that, in general, drug susceptibility toward BZ or NFX is highly variable among *T. cruzi* strain and depends on DTU genotype, parasite stage and possibly to some individual life trait history of each strain [65, 66]. Our previous studies showed that the mechanisms involved in natural drug resistance in *T. cruzi* differ from those involved in induced resistance, since that drug resistance in *T. cruzi* is a complex process involving different parasite stages, various metabolic pathways and the immune system of the host [11–13, 34, 40, 41]. Comparative analysis of the differential gene expression among susceptible and natural or clinically resistant *T. cruzi* isolates is difficult to investigate since that each strain presents its own genetic variability. Thus, there is very little information available on the biochemical mechanisms underlying drug resistance in field isolates of this parasite. The DE transcripts identified in our study can be further evaluated as potential molecular markers for drug resistance phenotype in field isolates.

The transcriptomic profile of *T. cruzi* revealed a robust set of genes from different metabolic pathways associated with the BZ-resistant phenotype. These DE transcripts can be further evaluated as molecular targets for new drugs against CD. The integration of different approaches, such as "-omics" technologies, cheminformatics, genetic manipulation and screening of potential inhibitors, is relevant to identify novel chemotherapeutic targets and compounds for CD. As a drug repositioning strategy, computational techniques could be employed for searching drugs that interact with DE proteins identified by our transcriptomic analysis. In addition, existing structural information about the potential molecular targets allows the application of molecular docking and structure-based drug design strategies for new drugs for CD treatment. Another important chemotherapeutic strategy for CD treatment is the use of a combination of compounds that inhibit different metabolic pathways of the parasite associated with the BZ-resistant phenotype identified in this study.

## Conclusions

In the present study we performed a transcriptome-level comparison of wild-type and BZ-resistant *T. cruzi* populations and generated a robust set of transcripts involved in different metabolic pathways associated with the BZ-resistant phenotype of the parasite. In general, our results agree with the postulate that *T. cruzi* resistance mechanisms are multifactorial and complex. Additionally, we were able to generate a high-quality transcriptome and identify transcripts known in the literature, such as APX and Fe-SOD. Functional analysis aided the identification of several important biological processes for the resistance phenotype, including antioxidant defense, inter- and intracellular molecular transport, changes in RNA processing and translation. These DE transcripts can be further evaluated as molecular targets for new drugs against CD. Further functional studies need to be performed to understand the role of hypothetical transcripts of unknown functions in the drug-resistant phenotype of *T. cruzi*.

## Supplementary Information

**Additional file 1: Table S1.** Characteristics of the reads that remained after the removal of low-quality data after Trimmomatic analysis.

**Additional file 2: Figure S1.** Expression graph of identified transcripts by each respective Log_2_FC found.

**Additional file 3: Table S2.** Enriched transcripts for biological process category with Gene Ontology-assigned terms.

**Additional file 4: Table S3.** Transcripts that were not enriched for biological process category with Gene Ontology-assigned terms.

**Additional file 5: Table S4.** Transcripts that were not enriched for biological process category without Gene Ontology-assigned terms.

**Additional file 6: Table S5.** Transcripts with unknown function without Gene Ontology-assigned terms.

## Data Availability

The datasets supporting the conclusions of this article are included within the article and its additional files. Sequences generated during the present study were deposited at the NCBI database under SRA Accession Numbers SRR22519198, SRR22519199, SRR22519200, SRR22519201, SRR22519202 and SRR22519203. BioSample Accession Numbers SAMN32007890, SAMN32007891, SAMN32007892, SAMN32007893, SAMN32007894, SAMN32007895 and BioProject PRJNA907829.

## Abbreviations

ADH: Alcohol dehydrogenase
APRT: Adenine phosphoribosyltransferase
APX: Ascorbate peroxidase
BP: Biological process
BZ: Benznidazole
CC: Cellular component
CD: Chagas disease
CDD: Conserved Domains Database
CPM: Counts per million
CyP: Cyclophilin
DE: Differentially expressed
eIF5A: Eukaryotic translation initiation factor 5A
Fe-SOD: Iron superoxide dismutase
GAF: Gene Association File
GAT3: Glycosomal ABC transporter 3
GFF: General feature format
GO: Gene Ontology
HT: Hexose transporters
MASP: Mucin-associated surface protein
MF: Molecular function
NFX: Nifurtimox
NTR-1: Nitroreductase type I
PCA: Principal component analysis
PGFS: Prostaglandin F synthase
RBP: RNA binding protein
RHS: Retrotransposon hot spot proteins
SOD: Superoxide dismutase
TAT: Tyrosine aminotransferase
