# Supplementary figures and images for: Transcriptomic analysis of benznidazole-resistant and susceptible Trypanosoma cruzi populations
Source: Parasit Vectors. 2023 May 22;16:167. doi: 10.1186/s13071-023-05775-4 (PMC10204194; doi:10.1186/s13071-023-05775-4)

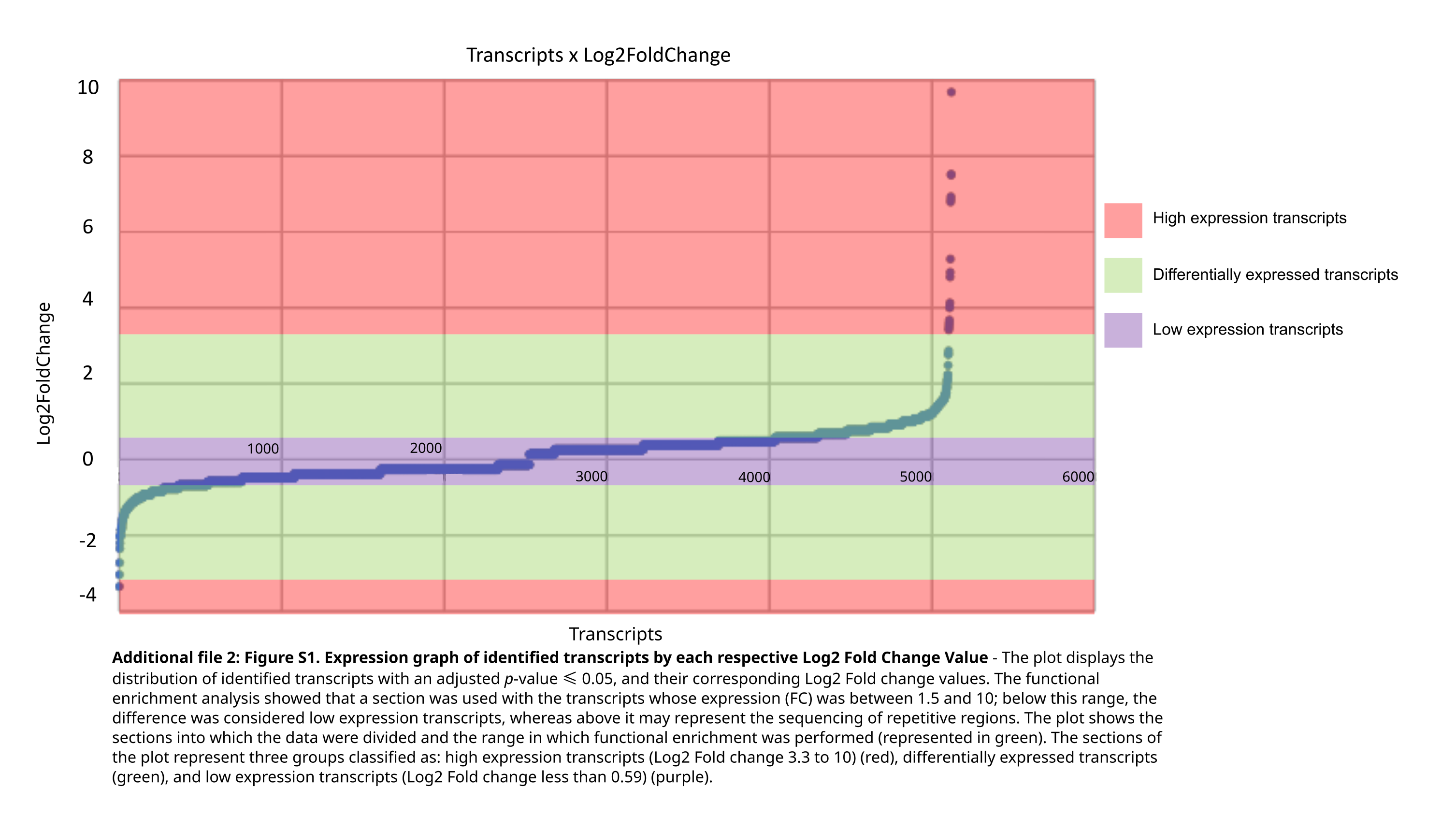

Supplement: Supplementary file 2 — Additional file 2: Figure S1. Expression graph of identified transcripts by each respective Log2FC found. [file 13071_2023_5775_MOESM2_ESM.tiff]
